# A serological survey of COVID-19 among predominantly aboriginal residents of a tourist island in southern Thailand

**DOI:** 10.1186/s41182-024-00617-0

**Published:** 2024-09-04

**Authors:** Supakorn Sripaew, Kameelah Yasharad, Dzerlina S. Rahari, Weiyan Feng, Zhenzhu Qian, Huynh Ngoc Thanh, Pei Li, Agus Fitriangga, Satiti Palupi Purwanto, Aye Nyein Phyu, Fangming Xianyu, Sombat Phadungvitvatthana, Wit Wichaidit, Ponlagrit Kumwichar, Virasakdi Chongsuvivatwong

**Affiliations:** 1 Department of Epidemiology, Faculty of Medicine, Prince of Songkla University, Hat Yai, Songkhla, 90110 Thailand; 2 Department of Family and Preventive Medicine, Faculty of Medicine, Prince of Songkla University, Songkhla, Thailand; 3 Office of Disease Prevention and Control Region 12, Songkhla, Thailand; 4 Satun Provincial Public Health Office, Satun, Thailand

**Keywords:** Ethnicity, Tourism, Chronic disease, Seroprevalence, COVID-19

## Abstract

**Background:**

The current survey describes the seroprevalence, history of coronavirus disease 2019 (COVID-19), and vaccination status among predominantly aboriginal residents on a tourist island in southern Thailand. This information can be translated into COVID-19 vaccination and control plans for this population.

**Methods:**

We implemented questionnaire interviews and collected blood samples from 249 residents of Lipe Island, Satun Province, in January 2022. We measured the anti-nucleocapsid protein and anti-spike (anti-S) receptor-binding protein levels of immunoglobulin (Ig) M and IgG. The differences in antibody levels among participants with different histories of vaccination and infection were analyzed using one-way analysis of variance with multiple comparisons.

**Results:**

During the 2-year pandemic period, no island residents with COVID-19 required hospitalization despite the high prevalence of hypertension (33.3%) and diabetes mellitus (21.7%). Approximately 18.8% of the participants reported a history of COVID-19 diagnosis. In total, 95.1% of the participants had a history of complete vaccination, of which 93.5% were seropositive. The anti-S IgG geometric means (geometric standard deviation) were 3945.8 (2.0), 829.8 (9.7) AU/mL, 789.9 (5.3) AU/mL, and 22.7 (7.1) AU/mL, respectively, in participants with a history of both COVID-19 diagnosis and complete vaccination (group 1), incomplete vaccination and subsequent COVID-19 diagnosis (group 2), complete vaccination but no previous infection (group 3), or neither previous COVID-19 and complete vaccination (group 4). Significant pairwise differences in anti-S IgG levels were found between certain groups (1 vs 3, 1 vs 4, 2 vs 4, and 3 vs 4).

**Conclusions:**

The high coverage of vaccination, high levels of population antibody titers, variable antibody levels among completely vaccinated non-infected residents, and high prevalence of non-communicable diseases (NCDs) suggested that the local health systems could control the pandemic. However, continuing surveillance, booster vaccinations, and NCD prevention programs were still required.

## Background

Thailand, a middle-income country in Southeast Asia, was the second country to report a case of coronavirus disease 2019 (COVID-19) in January 2020 [1]. The southern region of the country is particularly affected, as tourism is the main source of income in the region. Lipe Island, located in the Andaman Sea off the coast of Satun Province, is a world-famous beach and diving destination with a population of approximately 1300 people, mostly belonging to the *Urak Lawoi* (*Chao Lay*/*Orang Laut*) indigenous group, who traditionally earned their living as fishermen [2], but abandoned their subsistence economy lifestyle with the onset of tourism. The men work as boat drivers, while the women are homemakers [3]. These adaptations introduced susceptibility to non-communicable diseases (NCDs), similar to indigenous peoples elsewhere [4]. Furthermore, these adaptations and the increase in NCDs also made the population more susceptible to COVID-19 diagnosis [5] and mortality compared to those with more resources [6].

The *Urak Lawoi* are indigenous people who have inhabited islands in the Andaman Sea for hundreds of years [7] and can be considered part of the larger population of over 370 million indigenous persons, constituting approximately six percent of the world population [8]. During the COVID-19 pandemic, the burden of disease among indigenous people was exacerbated by their social and economic vulnerabilities, including income loss and high density of household members, which in turn resulted in poorer health outcomes compared to non-indigenous people [9, 10]. According to previous reports [11–13], extremely overcrowded housing was reported in Māori (New Zealand), Aboriginal and Torres Strait Islander (Australia), and Métis and Inuit (Canada) populations, which could make indigenous populations more prone to COVID-19 transmission [11]. In addition, limited healthcare access during the pandemic further exacerbates the burden of COVID-19 among indigenous people [9]. Even though public health authorities have made substantial efforts in vaccination programs, the lack of well-organized surveillance and reporting systems in indigenous communities presents gaps in COVID-19 control [9, 14–16].

In Thailand, COVID-19 vaccine roll-out had commenced in early 2021. In the first half of the year, three vaccines were first imported and distributed to the general population, including Lipe residents: a recombinant, replication-deficient chimpanzee adenovirus (ChAdOx1), an inactivated SARS-CoV-2 antigen (CZ02 strain), and an inactivated SARS-CoV-2 antigen (Tan-HB02 strain). With the continuous emergence of new SARS-CoV-2 strains, an mRNA encoding the viral spike protein of SARS-CoV-2 (Omicron XBB.1.5) was approved by the Food and Drug Administration in August 2021, and became one of the ministry-recommended vaccines [17, 18].

The first case of COVID-19 on Lipe Island was detected in June 2021, coinciding with the commencement of vaccination efforts by local health authorities. In August 2021, the incidence of COVID-19 in the island peaked, prompting the government to impose travel restrictions. After the island’s closure, the health authorities continued their efforts to vaccinate the island residents according to the ministry policy. In November 2021, the latest vaccine (ChAdOx1) batch was distributed to the residents, and approximately 90% of the population was reportedly vaccinated with at least two doses of either inactivated viral antigen, viral vector, or mRNA vaccines.

Transmission of COVID-19 in a community depends on the level of immunity in the population, which is determined by the depletion and repletion of susceptible individuals [19]. However, COVID-19 diagnoses are often asymptomatic [20], which undermines assessment based solely on reported cases and vaccination data. Furthermore, immunity levels may change over time after natural acquisition (i.e., infection) or vaccination, further complicating the assessment. Previous studies in Thailand had been conducted only among the general population and health care providers [21, 22] There was no baseline data on immunity levels for the local residents of Lipe Island, who are predominantly *Urak Lawoi* [23].

Owing to the importance of Lipe Island tourism to Satun’s economy, the local public health office has been under great pressure from residents, business owners, and the national government to reopen the island to tourism. However, such decisions must be balanced against the potential impact on the health of the island’s residents, particularly the *Urak Lawoi* people. A seroprevalence survey of the local population combined with self-reported information on COVID-19 vaccination and infection history can yield objective empirical data to support this important decision. The findings from such surveys can inform decision-makers regarding the level of immunity/vulnerability of the residents and also help in planning the future course of action to prevent and control the pandemic. Therefore, this study aimed to describe the seroprevalence and levels of antibodies against COVID-19 among the predominantly aboriginal *Urak Lawoi* residents of Lipe Island, Satun Province, southern Thailand.

## Methods

### Study design and setting

We conducted a cross-sectional study that included a questionnaire interview and serological survey of Lipe Island between January 14 and 16, 2022, after the pandemic in the country started to subside. The island has an area of 1.5 km [2], a population of approximately 1300, and a health center where primary care, including vaccination, is provided. During the COVID-19 outbreak, a system was established to isolate infected patients and quarantine individuals in close contact. Local hotels and schools on the island were used for such isolation. If patients needed urgent or emergent care, they were transferred to the mainland hospital by speedboat; however, no severe cases were detected. A recent health region survey over seven provinces adjacent to Satun province, where the current survey was conducted, showed that B.1.1.529 (Omicron) was the most common variant in the region, accounting for 56.4% of the 3215 randomly selected samples. Due to the island’s closure, the island vaccination program was scheduled by the Department of Disease Control and Prevention, Health Region 11, Ministry of Public Health, to immunize the residents as early as in the early phase of the epidemic. According to the policy [17, 18], only certain vaccines were available at the time: (1) ChAdOx1 viral vector vaccine, (2) CZ02 strain inactivated vaccine, (3) Tan-HB02 strain inactivated vaccine and (4) mRNA (Omicron XBB.1.5) vaccine, of which their administrations were scheduled based on the national batch-of-vaccine-supply policy.

### Study population and selection of study participants

The study population included all residents of Lipe Island who were listed in the local health office registry. The inclusion criteria were as follows: (1) age 18 years or older and (2) ability to communicate in Thai, Malay, English, or Burmese.

We calculated the sample size based on the assumption that the prevalence was 83.0%, with a limit of 95% ± 5.0% from the estimate. A total of 217 respondents were required to address the study’s objectives.

To select our study participants, we requested a population registry of the island from a local public health office. We then used a spreadsheet to select the required number of eligible participants using simple random sampling and informed the health office of the list of sampled residents. The health office then coordinated with the local health center to contact the sampled residents and invite them to the data collection session at a designated time and place.

### Data collection and serological testing

After a pilot study in a similar coastal community, questionnaire interviews were conducted by Thai graduate students, one faculty member, and six research assistants after obtaining written informed consent. Data were directly entered into a server using a web-based application (KoboToolbox, available at Kobotoolbox.org). Investigators then exported the entered data, which contained demographic and health characteristics (age, sex, education, occupation, income, household size, smoking status, and medical conditions), vaccination status, history of COVID-19 diagnosis, and the participant identification number to a spreadsheet file.

After the interview, licensed medical technologists and two registered nurses collected cubital venous blood from participants who consented to the blood draw. Investigators separated whole blood from each participant into three aliquots and stored the aliquots at 3–5 °C at a nearby community health center. Medical technologists then transported all refrigerated blood samples to the Office for Disease Prevention and Control Region 12 within 72 h after collection, where antibody levels were measured. The technologists were blinded to the interview data, and the serological test results were exported to another spreadsheet file with the project identification numbers.

We used anti-spike receptor-binding protein (anti-S RBD) and anti-nucleocapsid protein (anti-N) to describe serological characteristics of the island residents. Anti-S test can aid the evaluation of individual immune status (its positivity) or monitoring antibody response (its quantity) after infection or vaccination. Anti-N was selected as a supplement for the anti-S test as it provides additional information about the plausibility of previous exposure to inactivated vaccines or previous COVID-19 infections rather than exposure to other vaccine types. [24] Thus it could help distinguish between infection or vaccination exposure in seropositive individuals. According to the national guideline on COVID-19 vaccination for the situation in 2021, complete vaccination was defined as status of having vaccinated with at least two doses of any vaccines.

Laboratory scientists quantified anti-S immunoglobulin G (IgG) levels in arbitrary units (AU/mL) and assessed the positivity of anti-S IgM (Architect i2000SR System, Abbott Laboratories Abbott Park, Illinois, United States) and IgG (SEMI-QUANT for use with Architect, Abbott Laboratories Abbott Park, Illinois, United States) using the chemiluminescent microparticle immunoassay technique. Anti-N IgM and IgG positivity were assessed using the EUROIMMUN ELISA test (PerkinElmer Waltham, Massachusetts, United States). IgM positivity indicates recent exposure to the antigen, and IgG positivity indicates past exposure. All the tests were claimed to be sensitive to detect antibodies developed after the exposure of any circulating severe acute respiratory syndrome coronavirus 2 (SARS-CoV-2) variants at the time. Anti-S IgG levels stabilize 6 months after SARS-CoV-2 infection [25], while anti-N IgG levels significantly decline 6 months post-infection [26]. After receiving the laboratory results, members of the data collection team reported the laboratory findings to consenting participants.

### Statistical analysis

After data cleaning, we merged the interview data with the laboratory results based on the project identification numbers. We used descriptive statistics to summarize the characteristics of the study participants and serological test results in each subgroup. Hypothesis testing between two categorical variables was performed using the Chi-square or Fisher’s exact test. For continuous variables, such as log-transformed antibody titers, one-way analysis of variance (ANOVA) was performed with a significance level set at < 0.05. For variables with statistically significant ANOVA test results, post hoc (Bonferroni) tests were performed to compare all pairs of transformed means with multiple comparison adjustments. R software version 4.1.0, with epicalc, lubridate, stringr, and ggplot2 packages, was used for all analyses.

## Results

Among 283 eligible participants, 249 participated in the interviews. Among the participants, four refused to undergo blood tests; thus, seroprevalence data were available for 245 participants, and 93.5% were seropositive (Table 1). Sixteen participants were seronegative, suggesting they had been neither infected nor vaccinated. Overall, 97.7% and 41.1% of seropositive individuals had anti-S and anti-N positive (including borderline results), respectively. IgM positivity was relatively low in the study participants (3.7% and 12.3% for anti-N IgM and anti-S IgM, respectively) compared with positive IgG status (31.7% and 93.4% for anti-N IgG and anti-S IgG, respectively). Among those who were seropositive, the majority had only IgG against the spike protein, which indicated that they were vaccinated with either the mRNA or viral vector vaccine, rather than vaccinated with inactivated vaccines or had previous COVID-19 infections. The second most common positive group had positive IgG against both spike and nucleocapsid proteins. This group could have been exposed to either an inactivated virus vaccine or natural infection, with or without vaccination.

**Table 1:**
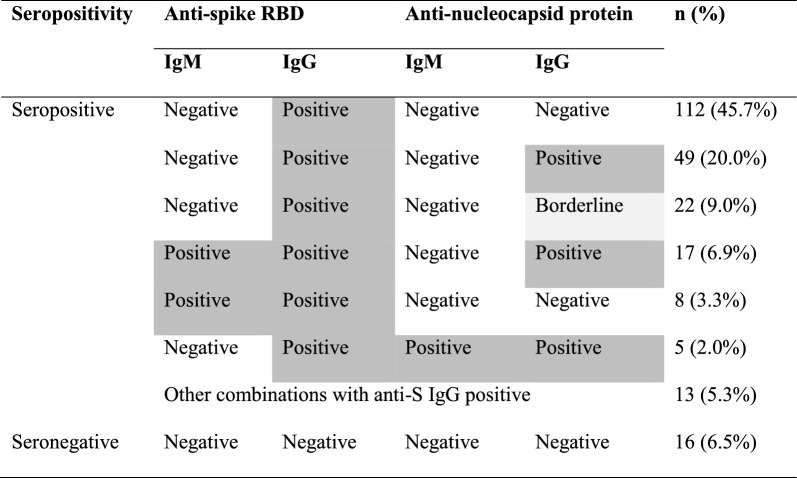
Seroprevalence of the study participants

Positive and borderline results are colored dark and light grey, respectively. RBD, receptor binding domain

The majority of the participants had less than a high school education and were unemployed or worked in class 1 occupations (laborer, agriculture or fishery) (Table 2). However, compared to seropositive participants, seronegative participants included a higher proportion of older persons, males, smokers, low-income persons, and those who never traveled to the mainland since the start of the COVID-19 pandemic. The majority of participants were Thai; there were only three non-Thai nationalities, two of whom were Burmese, and one of them was American. Approximately half of the participants had a history of at least one of the following diseases: hypertension (33.3%), hyperlipidemia (25.7%), diabetes mellitus (21.7%), asthma (6.4%), or chronic kidney diseases (3.2%). However, seronegative participants also had a higher prevalence of chronic disease compared to seropositive participants.

**Table 2:** General characteristics of the study participants

Characteristics (*N* = 245)	Seropositive (*n* = 229)	Seronegative (*n* = 16)
Age group in year (*n*, %)		
< 30	48 (21.0)	2 (12.5)
30–39	54 (23.6)	4 (25.0)
40–49	52 (22.7)	1 (6.2)
50–59	41 (17.9)	3 (18.8)
60 or more	34 (14.8)	6 (37.5)
Female (*n*, %)	163 (71.2)	8 (50.0)
Current smokers (*n*, %)	33 (14.4)	4 (25.0)
Religion (*n*, %)		
Buddhism	197 (86.8)	15 (93.8)
Islam	14 (6.2)	1 (6.2)
Christianity	14 (6.2)	0 (0)
Others	2 (0.9)	0 (0)
Marital status (*n*, %)		
Married	190 (82.9)	15 (93.8)
Single	29 (12.7)	1 (6.2)
Divorced/ widowed/ separated	10 (4.4)	0 (0)
Education level (*n*, %)		
No formal education	17 (7.4)	2 (12.5)
Primary school	130 (56.8)	10 (62.5)
Junior high school	57 (24.9)	3 (18.8)
High school and higher	25 (10.9)	1 (6.2)
Occupation (*n*, %)		
Class 1 occupation (laborers, agriculture/fishery)	87 (38.0)	6 (37.5)
Class 2 occupation (vendors, service providers)	8 (3.5)	0 (0)
Class 3 occupation (business owner, civil servant, private sector employee, and independent professions)	82 (35.8)	3 (18.8)
Unemployed/unpaid work	52 (22.7)	7 (43.8)
Personal monthly income (*n*, %)		
< 5000 Baht	97 (42.4)	10 (62.5)
5000 to 10,000 Baht	67 (29.3)	3 (18.8)
10,001 to 20,000 Baht	30 (13.1)	1 (6.2)
20,001 to 30,000 Baht	2 (0.9)	0 (0)
> 30,000 Baht	7 (3.1)	0 (0)
Not sure/unstable/no answer	26 (11.4)	2 (12.5)
Household size (median, IQR)	5 (3,6)	4 (2.8,5)
Relationship with the head of household (*n*, %)		
Respondent is the head of household	117 (51.1)	10 (62.5)
Wife or husband or partner	61 (26.6)	4 (25.0)
Others	51 (22.3)	2 (12.5)
Traveling to the mainland (*n*, %)		
Never	91 (39.7)	9 (56.2)
< 1 time/month	110 (48)	5 (31.2)
At least once a month	28 (12.2)	2 (12.5)
Traveling to other provinces (*n*, %)	36 (15.7)	1 (6.2)
Medical conditions (*n*, %)		
Diabetes	49 (21.4)	3 (18.8)
Hypertension	74 (32.3)	6 (37.5)
Hyperlipidemia	54 (23.6)	7 (43.8)
Treated pulmonary tuberculosis	5 (2.2)	1 (6.2)
Chronic kidney disease	7 (3.1)	1 (6.2)
Chronic lung disease	23 (10.0)	4 (25.0)
Other chronic diseases	39 (17.0)	5 (31.2)
One or more of the above conditions	126 (55.0)	12 (75.0)

Table 3 describes vaccination regimen among the study participants (*n* = 243). Most participants received complete vaccination. Only seven and four participants were not vaccinated and vaccinated with only one vaccine, respectively. The most common type of vaccine regimen was an alternate regimen involving an inactivated vaccine (CZ02 strain) and a viral vector vaccine (ChAdOx1) (25.5%). Only two participants had received the mRNA vaccine as their third dose. Among the vaccinated persons, the longest approximated time since the last vaccination was 31 weeks (4.5%), while most of the participants were vaccinated 3–6 months prior to the serological testing. Considering potential anti-N seropositivity, approximately half and one-fifth of the participants were vaccinated with inactivated vaccines (A or B) longer than and within the previous 6 months, respectively.

**Table 3:** Vaccination regimen of the study participants

Number of doses	Type of vaccine received			n (%)
	1st dose (duration*)	2nd dose (duration*)	3rd dose (duration*)	
0	NA			7 (2.9)
1	A (10)			4 (1.7)
2	B (25)	A (20)		62 (25.5)
	C (18)	C (15)		40 (16.5)
	A (32)	A (20)		20 (8.2)
	B (35)	B (31)		11 (4.5)
	Other			4 (1.6)
3	B (35)	B (31)	A (10)	43 (17.7)
	B (25)	A (20)	A (10)	11 (4.5)
	C (18)	C (15)	A (10)	4 (1.6)
	Other			7 (2.9)

A = single recombinant, replication-deficient chimpanzee adenovirus (ChAdOx1)

B = inactivated SARS-CoV-2 antigen (CZ02 strain)

C = inactivated SARS-CoV-2 antigen (Tan-HB02 strain)

NA = not applicable

Other group includes various other combination of majorly A, B, and C vaccines. Only two individuals had received mRNA vaccine (Omicron XBB.1.5) for their third vaccinations

*Approximated duration of vaccination in weeks since the vaccination date; the time wholly depended on the vaccination scheduled by the island’s authorities, which had required the participants to be vaccinated only in a certain period

Within a 2-year period since the beginning of the pandemic, with 18.7% of the island residents diagnosed with COVID-19, there had not been any hospitalized patients. Participants with two or more vaccine doses were more likely to be seropositive (Table 4). Universal seropositivity was generally found among those with two or more vaccine doses, except for participants with no history of COVID-19 diagnosis or close contact with COVID-19 cases. A minority group (approximately 10%) was seronegative. All participants who received the three doses of vaccination were seropositive.

**Table 4:** Seroprevalence according to status of vaccination and previous infection

Characteristics	Seropositive (*n*, %)	Seronegative (*n*, %)	*p* Value*
History of COVID-19 diagnosis (*n* = 46)			
Incomplete vaccination	4 (80%)	1 (20%)	0.109
2-dose vaccination	26 (100%)	0 (0%)	
3-dose vaccination	15 (100%)	0 (0%)	
No history of COVID-19 diagnosis (*n* = 199)			
History of close contact (*n* = 43)			
Incomplete vaccination	2 (66.7%)	1 (33.3%)	0.070
2-dose vaccination	28 (100%)	0 (0%)	
3-dose vaccination	12 (100%)	0 (0%)	
No history of close contact (*n* = 156)			
Incomplete vaccination	1 (25%)	3 (75%)	< 0.001
2-dose vaccination	98 (89.9%)	11 (10.1%)	
3-dose vaccination	43 (100%)	0 (0%)	

*Fisher’s exact test

**Fig. 1:**
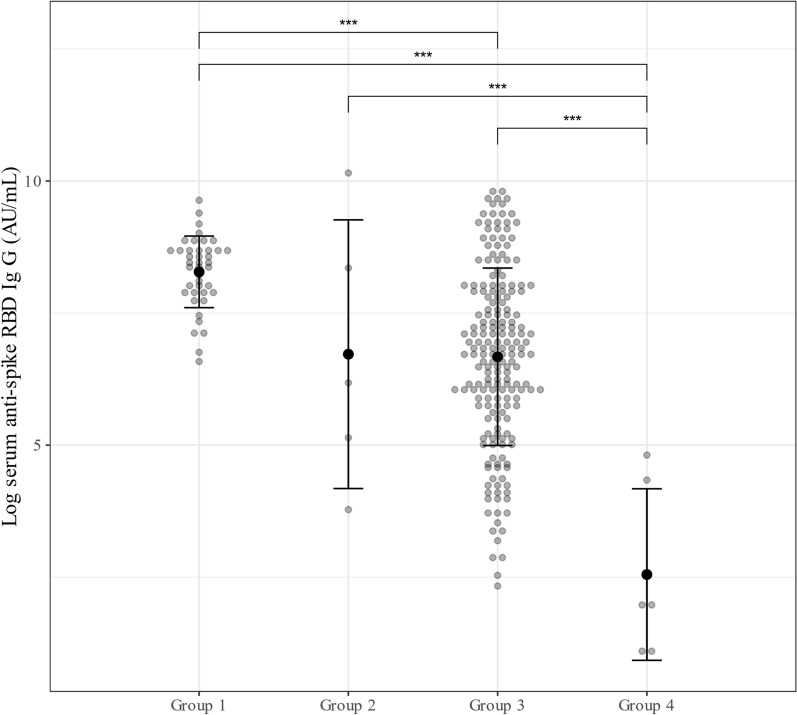
Distribution of anti-spike receptor-binding domain IgG titer (in natural logarithmic scale) by history of COVID-19 diagnosis and vaccination status. Group 1: history of COVID-19 diagnosis, complete vaccination; Group 2: history of COVID-19 diagnosis, incomplete vaccination; Group 3: no history of COVID-19 diagnosis, complete vaccination; Group 4: no history of COVID-19 diagnosis, incomplete vaccination, thick dot and whiskers denote group mean ± SD. *** Denotes significant difference between groups with *p* < 0.001

A plot of the log-transformed anti-S receptor-binding domain IgG titer versus the history of COVID-19 diagnosis and vaccination status (incomplete vs. complete) showed significant differences in titer levels between groups, except for the differences between Groups 1 and 2 and between Groups 2 and 3 (Fig. 1). Calculation of geometric means showed that those with a history of COVID-19 diagnosis and complete vaccination had the highest geometric mean level of anti-S IgG titer, and those with no history of COVID-19 diagnosis and incomplete vaccination had the lowest geometric mean level (Table 5).

**Table 5:** Geometric mean levels of anti-s IgG (AU/mL) by history of COVID-19 diagnosis and vaccination status

History of COVID-19 diagnosis and vaccination status	Geometric mean of anti-s IgG level (geometric SD) (AU/mL)
Group 1: history of COVID-19 diagnosis, complete vaccination (*n* = 41)	3945.8 (2.0)
Group 2: history of COVID-19 diagnosis, incomplete vaccination (*n* = 5)	829.8 (9.7)
Group 3: no history of COVID-19 diagnosis, complete vaccination (*n* = 192)	789.8 (5.3)
Group 4: no history of COVID-19 diagnosis, incomplete vaccination (*n* = 7)	22.7 (7.1)

**Fig. 2:**
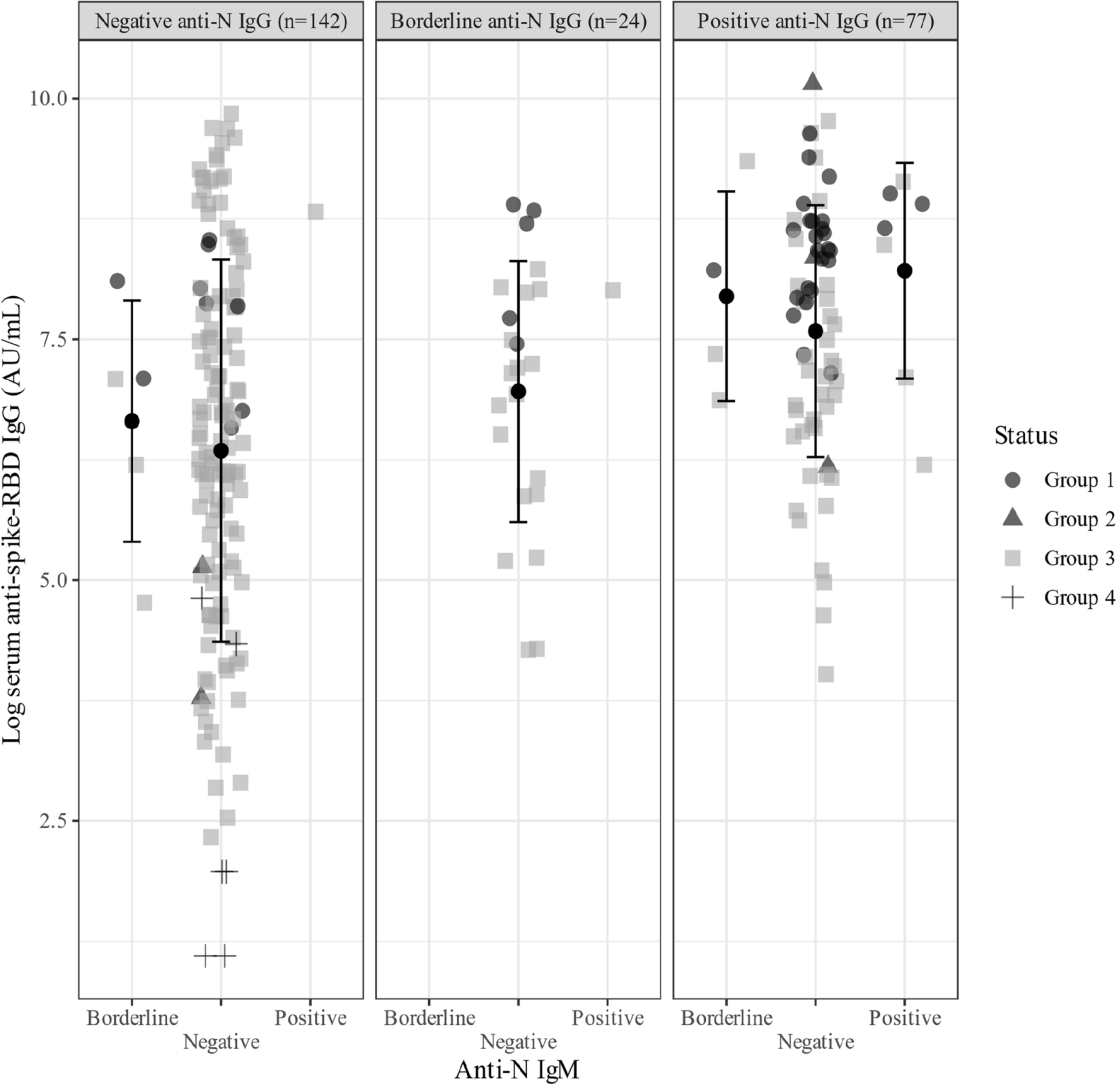
Distribution of anti-spike receptor-binding domain IgG titer (in natural logarithmic scale) by anti-nucleocapsid protein (IgM and IgG), history of COVID-19 diagnosis, and vaccination status. Group 1: history of COVID-19 diagnosis, complete vaccination; Group 2: history of COVID-19 diagnosis, incomplete vaccination; Group 3: no history of COVID-19 diagnosis, complete vaccination; Group 4: no history of COVID-19 diagnosis, incomplete vaccination, thick dot and whiskers denote group mean ± SD

Figure 2 illustrates levels of log-transformed anti-S IgG stratified by anti-N IgM and IgG. The group of participants who reported previous COVID-19 diagnosis, with most participants completely vaccinated (89.1%), had relatively higher anti-S IgG levels than the other groups. Almost all members of the group with negative anti-N antibodies had complete vaccination but did not report previous infection (group 3). These participants had higher variability of anti-S IgG than the other groups. A higher proportion of participants with a history of COVID-19 diagnosis had positive anti-N IgM (33.3%) and positive anti-N IgG (39.0%) than other anti-N statuses (9.8%).

## Discussion

We conducted a seroprevalence survey combined with interviews of residents (most of whom are indigenous people) of Lipe Island, a tourist destination in southern Thailand. We found a relatively high prevalence of NCDs among study participants. The majority of the participants also reported receiving two or three doses of the COVID-19 vaccine, whereas a history of COVID-19 diagnosis was relatively uncommon. Anti-S IgG was found in approximately nine-tenths of the participants, half of whom did not have anti-N IgG. Seropositivity was strongly associated with the receipt of two COVID-19 vaccine doses. The findings also showed that participants who had a history of COVID-19 diagnosis were more likely to have positive anti-N IgM and IgG than the non-infected persons. Apart from that, a relatively more variable anti-S IgG level was found in the group where residents were completely vaccinated and non-infected.

The current serological survey took place while the second wave of COVID-19 on the island was subsiding. The island vaccination program had been deployed for approximately 2 months, and almost all island residents had been vaccinated. The overall seroprevalence in this study was remarkably higher than that in other larger islands demonstrated in previous studies. A bigger island (with approximately fifty thousand inhabitants) in Denmark [27] had remarkably smaller seroprevalence (93.5% versus 0.7%) at the beginning of the pandemic (April 27–May 1, 2020) when vaccines were still under development. In another comparably populated and partially vaccinated (42% and 10% having received at least 2 doses inactivated SARS-CoV-2 and ChAdOx1 vaccine, respectively, of which their last dose was approximately 7 weeks prior to the serological testing) island (3612 residents) in Brazil, where a serological survey took place at approximately 5 months after the vaccine roll-out, the seropositive rate was 53.6% [28]. However, our dose–response relationship in seroprevalence across vaccination-infection groups was consistent with that in a study from Cyprus conducted in the second half of 2021 when the national vaccination program had been deployed for 6 months, and 54.2% of the participants were vaccinated [29]. The anti-spike-receptor binding domain (RBD) IgG levels among individuals who were not vaccinated and had never been diagnosed with COVID-19 were significantly lower than those in the other groups. Nevertheless, the levels of the IgG in the current study were almost at one-fifth of that of the Cyprians. According to previous studies [30, 31], anti-spike-RBD IgG levels peak approximately 4 weeks after the second injection (i.e., complete vaccination) and significantly decline at 3–6 months after vaccination to levels comparable with those in individuals with immunity acquired from natural infection. Thus, the antibody levels suggested that residents with complete vaccination were recently vaccinated.

The current study found that approximately 4% of seropositive individuals were anti-nucleocapsid protein IgM positive, and a history of COVID-19 diagnosis was relatively common in the participants who had positive anti-N IgM and anti-N IgG than in the other groups. Accordingly, a recent COVID diagnosis, which was likely represented by anti-N positivity [32], might have been relatively small in the sample. In addition, we found that the overall seropositivity was positively associated with vaccination rather than a history of COVID-19 diagnosis, with a clear dose–response relationship. Similar results were also found abroad and in a study based in Thailand [31, 33]. Further, we found that almost all the persons who had antibodies against spike protein were likely persons who had completed vaccination rather than persons who had COVID-19 diagnosis. Therefore, we assessed that the seropositivity and anti-S antibody mainly represented high vaccination coverage. The 95.1% prevalence of complete vaccination in this study was higher than in studies in Australia^15^ and Canada^16^. The high coverage on this tourist island could be partly attributed to the strong policy of the government and the enthusiasm of the local population to maximize vaccination coverage to ensure the reopening of the country to international tourism.

According to a previous study [34], circulating anti-S-RBD and anti-N antibody were significantly correlated with the immune neutralizing activity. The second figure shows relatively higher proportions of individuals who had previous COVID-19 infection with complete vaccination (i.e., hybrid immunity) than persons who only had vaccination (without reported COVID-19 diagnoses) in a group where both anti-N IgM and/or IgG were positive (contributed approximately 29% of the sample). Our serological results combined with self-reports could supplement a previous finding of high magnitude and durability of COVID-19 protection in persons with hybrid immunity [35]. Furthermore, the anti-S level of the participants with hybrid immunity were relatively higher than that of the participants who were completely vaccinated but did not report previous COVID-19 diagnoses. This difference was also observed in another study, which also revealed that individuals who were both completely vaccinated and previously infected by SARS-CoV-2 had slower antibody decline than those who were solely vaccinated [36]. Apart from that, higher variability of anti-S IgG in persons who solely had complete vaccination than in the other groups, and variability of vaccination regimen (mainly inactivated non-Omicron SARS-CoV-2 variant vaccines while the most common variant was Omicron) highlights the importance of long-term monitoring of new infections despite high vaccination coverage in the island residents. Re-opening the island would potentially exponentiate the number of new COVID-19 cases while the existing immunity wanes [37]. Thus, we suggested that local public health authorities should consider continuing disease surveillance and organizing booster vaccination programs to maintain protection against the virus among island residents [38].

The prevalence of NCDs was higher in the study population than in the general population of Thailand [39]. Economic development, including tourism, has been shown to change the lifestyles of indigenous people, making them more prone to NCDs [40]. The local population in this study was protected from the pandemic; however, they are still highly vulnerable to the long-term effects of NCDs and decaying vaccine efficacy [41], which require different solutions from those for the pandemic. NCDs are likely to be aggravated once the tourism industry re-opens on this island. The high prevalence of NCDs, high coverage of vaccination, high levels of population antibody titers, and low levels of complications from COVID-19 suggest that the local health systems have been on target for pandemic control but not for NCDs. Control of COVID-19 should be continued in parallel with the improvement of NCD prevention.

This study was the first to assess the COVID-19 seroprevalence in a predominantly indigenous population living in a tourist resort island in Thailand. The findings provided insights into the seroprevalence and antibody profile in Aboriginal people who are biologically and socio-behaviorally susceptible to infection. Nevertheless, several limitations should be considered when interpreting our study findings. First, participants were predominantly female, with relatively few working-age participants. Caveats should be considered when generalizing the information to the population of the entire island. Second, our calculated sample size might not have yielded adequate statistical power to assess the association between seropositivity, vaccination, and history of diagnosis with COVID-19. Thus, we could not rule out chance as the best explanation for the observed findings. Lastly, we only investigated individuals’ antibody statuses, which are indirect measurements for individual protection against COVID-19; antibody levels in the study population may wane over time, and new strains of SARS-CoV-2 may emerge. Thus, the high seroprevalence at the time of study may not necessarily imply full and sustained protection against future SARS-CoV-2 infections.

## Conclusion

We observed high seroprevalence rates, likely attributable to the vaccination program, alongside a low incidence of COVID-19 cases during the outbreak on Lipe Island. However, antibody levels among the completely vaccinated residents without a history of COVID-19 varied, and there was a comparably high prevalence of chronic diseases among the residents. This serological survey can help explain the relatively low level of COVID-19 health burden in this population and guide control policy. This approach can be applied to similar populations.

## Data Availability

Fully de-identified data are available upon written request to the corresponding author.

## Abbreviations

Anti-S RBD: Anti-spike receptor-binding protein
Anti-N: Anti-nucleocapsid protein
NCD: Non-communicable disease
